# A study on the application of topic models to motif finding algorithms

**DOI:** 10.1186/s12859-016-1364-3

**Published:** 2016-12-22

**Authors:** Josep Basha Gutierrez, Kenta Nakai

**Affiliations:** 1 Department of Computational Biology and Medical Sciences, Graduate School of Frontier Sciences, The University of Tokyo, 277-8561 Chiba, Japan; 2 Human Genome Center, The Institute of Medical Science, The University of Tokyo, 4-6-1 Shirokane-dai, Minato-ku, 108-8639 Tokyo, Japan

## Abstract

**Background:**

Topic models are statistical algorithms which try to discover the structure of a set of documents according to the abstract topics contained in them. Here we try to apply this approach to the discovery of the structure of the transcription factor binding sites (TFBS) contained in a set of biological sequences, which is a fundamental problem in molecular biology research for the understanding of transcriptional regulation. Here we present two methods that make use of topic models for motif finding. First, we developed an algorithm in which first a set of biological sequences are treated as text documents, and the k-mers contained in them as words, to then build a correlated topic model (CTM) and iteratively reduce its perplexity. We also used the perplexity measurement of CTMs to improve our previous algorithm based on a genetic algorithm and several statistical coefficients.

**Results:**

The algorithms were tested with 56 data sets from four different species and compared to 14 other methods by the use of several coefficients both at nucleotide and site level. The results of our first approach showed a performance comparable to the other methods studied, especially at site level and in sensitivity scores, in which it scored better than any of the 14 existing tools. In the case of our previous algorithm, the new approach with the addition of the perplexity measurement clearly outperformed all of the other methods in sensitivity, both at nucleotide and site level, and in overall performance at site level.

**Conclusions:**

The statistics obtained show that the performance of a motif finding method based on the use of a CTM is satisfying enough to conclude that the application of topic models is a valid method for developing motif finding algorithms. Moreover, the addition of topic models to a previously developed method dramatically increased its performance, suggesting that this combined algorithm can be a useful tool to successfully predict motifs in different kinds of sets of DNA sequences.

**Electronic supplementary material:**

The online version of this article (doi:10.1186/s12859-016-1364-3) contains supplementary material, which is available to authorized users.

## Background

Sequence motifs are short patterns that occur in DNA with certain frequency and that often have some sort of biological distinct function. In most cases, that function is to serve as a binding site for proteins. When these proteins are transcription factors (TF), the corresponding motifs are called transcription factor binding sites (TFBS). Knowing these TFBS gives a better understanding of how transcriptional regulation works, and therefore the discovery of TFBS is one of the most fundamental problems in molecular biology research [1, 2]. Historically, a wide variety of methods have been applied to this problem, computational methods being currently the prevailing approach. The computational problem consists of discovering motifs by searching for overrepresented (and/or conserved) DNA patterns in sets of functionally related genes, such as genes with similar functional annotation or genes with similar expression patterns. The number of different computational approaches to tackle this problem is constantly growing as computational techniques evolve. One of the most recent techniques, which, to the best of our knowledge, to this date has not yet been applied to motif discovery, is known as *topic models* [3].

### Topic models

Topic models are statistical algorithms, based on natural language processing and machine learning, which try to discover the structure of a set of documents according to the abstract topics contained in them by hierarchical Bayesian analysis [4]. These algorithms allow examining a set of documents and determining the existing topics and their distribution among the documents based on the statistical properties of the words of a specific vocabulary in each one of them. *where L* Application of topic models to the motif finding problem.

**Fig. 1:**
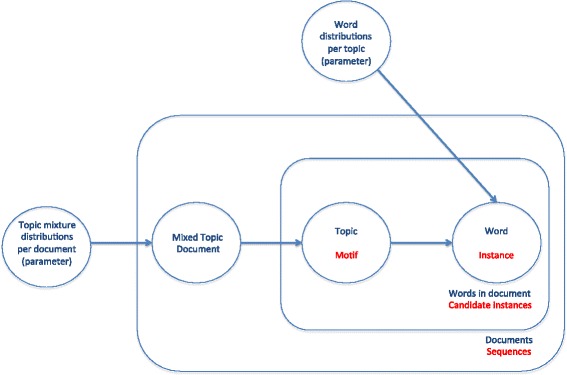
Representation of a topic model adapted to the motif finding problem. Representation of a topic model adapted to the motif finding problem. This figure shows the basic structure of a topic model (in this case, a LDA). The terms specific for the case of the motif finding problem are stated in red under the original ones in blue, showing that the motif finding problem can be represented by a topic model by describing the DNA sequences as documents, the instances of each given motif as words in those documents, and the motifs as clusters of words or topics

As far as we know, there is no literature about the application of topic models to motif finding algorithms. The first method here proposed tries to fill that gap and prove that topic models are a suitable method to the motif finding problem. In order to do so, it represents genetic sequences as documents and the k-mers contained in them as words, so that the patterns shown among these k-mers would be considered as motifs. Figure 1 shows a graphic representation of a topic model and how our algorithm would adapt to it.

The algorithm, as a topic model, would therefore examine a set of sequences to determine the hidden structure of the patterns contained in it. As this is totally consistent with the motif finding problem, it seems likely that the algorithm should be able to correctly discover motifs.

### Addition of topic models to a previously developed algorithm (Statistical GA)

Previously to this study of topic models applied to the motif finding problem, we developed another algorithm with the structure of a GA, which used statistical coefficients as a fitness measurement [5]. From this point, we will refer to this algorithm as the *Statistical GA* algorithm. The Statistical GA algorithm was proven to effectively find overrepresented motifs in sets of sequences. However, it had the main drawback of reporting an excessive number of false positives. Along with the algorithm based entirely on topic models, in this study we also research how the use of topic models can be applied to improve the previously developed algorithm and reduce the number of false positives.

## Methods

### How topic models work

The main problem, from a computational point of view, of topic modeling is to infer a concealed topic structure from the examination of the documents.

A topic is formally defined as a multinomial distribution over a fixed vocabulary. In other words, topic models consider that a document could, conceptually, be generated from a set of topics, each one of them being a set of words related to that topic. So that, to create a document, the words would be selected iteratively from the topics that we desire to appear in it. For example, if we want a document that is two thirds about stem cells and one third about cancer, we would create two topics (stem cells and cancer) as sets of words typically related to them, and then construct the document by selecting two thirds of the words from the *stem cells* set and one third from the *cancer* set.

Topic modeling consists of reversing this conceptual approach, considering that the topics of a document (or a set of documents) can be inferred from the proportions of the words contained in them.

The intuition behind this algorithm is that all of the documents in the collection share the same set of topics, but each one of them in a different proportion, which is reflected in the distribution of the different words among them.

The inputs of a topic model are a set of *N* documents (*d*_*1*_ *, …, d*_*N*_) and the number of topics *K* that are expected to be contained in the documents. For each one of the documents *d*_*i*_ to be analyzed, the most basic algorithm would process the words in a two-stage process.

1. Choose a random distribution of the document over the topics (*t*_*1*_ *, …, t*_*K*_).
2. For each word *w*_*j*_ in the document:
  - Choose a random topic *t*_*r*_ from the distribution over topics previously generated.
  - Once *w*_*j*_ is assigned, for each one of the topics *t*_*m*_ in the current set of topics, compute the proportion of words in the document *d*_*i*_ that are currently assigned to the topic *t*_*m*_, *P(t*_*m*_ *|d*_*i*_ *)*, and the proportion of assignments to the topic *t*_*m*_ over all of the documents that come from the word *w*_*j*_ , *P(w*_*j*_ *|t*_*m*_ *)* and then reassign *w*_*j*_ to the topic that gives the best probability *P(t*_*m*_ *|d*_*i*_ ) * P(w_*j*_ *|t*_*m*_ *)*.

A stable set of assignments will be reached after repeating the above steps for several iterations.

The benefit of the use of topic models is that they offer an automated solution to the organization and annotation of large text archives. However, this is not their only utility, and they can be applied to many other fields, such as the subject in question here, bioinformatics.

### Creating a motif finding algorithm based on topic models from scratch

#### Algorithm structure

The first problem that arises when adapting a topic model to the motif finding problem comes from the idea that, whereas the words in a text document are clearly separated by spaces, in the case of a genetic sequence a mechanism to select the k-mers that will form the vocabulary must be defined.

A typical topic model, as a first step, usually creates a vocabulary from the words in the documents by discarding meaningless words (in terms of determining a topic), such as “the” or “of” in documents written in English, as well as words that are not repeated frequently, since in both cases they would not help to find the hidden topics and they would instead add noise to the algorithm. Again, this is consistent with a motif finding algorithm, so in this case an initial vocabulary would need to be created similarly, but in this case by selecting k-mers that are overrepresented in the set of sequences.

From this a new problem arises, which is the impossibility to select all of the possible overrepresented patterns in a reduced amount of time. In order to deal with that, a genetic algorithm (GA) [6] structure was chosen as the basis of the algorithm here presented, being the topic model the approach for selecting the best possible solutions in the fitness function.

#### Algorithm implementation

The method here proposed is a heuristic algorithm, that is, it gives an approximate (not necessarily optimal) solution, and it is also stochastic, so that each time it is run with the same set of sequences it will likely produce different results. It searches only for ungapped motifs, so that patterns which contain gaps might be predicted split into several separate motifs. Also, in contrast with other motif finding algorithms, which usually suppose that there is at least an instance of the motifs in every sequence of the data set, it makes no assumptions about how the motifs are distributed among the sequences.

The algorithm is implemented as a classic GA. In other words, it starts by creating a population of possible solutions (individuals) for our problem and then it iterates over them, keeping the best (fittest) solutions of every iteration, discarding the worst ones, and creating new solutions based on the fittest ones for the following iteration, until an optimum solution is found or a given number of iterations is reached.

Therefore, the only aspects that need to be defined are how the population is represented, how it is evaluated (fitness), how the fittest individuals are selected in every iteration, how new individuals are generated by the surviving ones (crossover, mutation) and when the algorithm will stop iterating and report a final solution (or set of solutions).

#### Representation

Each individual of the population is a set of *m* k-mers which can be contained in any of the sequences of the data set. The k-mers can be of any length between a minimum and a maximum passed as a parameter. The initialization works as follows:

Given a set of sequences, a minimum k-mer length *k*_*min*_, a maximum k-mer length *k*_*max*_, a minimum number of repetitions for each k-mer in the data set *c*_*min*_, a population size *N*, and a number of words per individual in the population *n*. For each one of the *N* individuals, iterate until an initial set of *n* k-mers is reached:

1. Choose a random word length *k* within the range *k*_*min*_ *: k*_*max*_.
2. Choose a random sequence from the data set.
3. Choose a random position *p* in that sequence between 0 and *l - k*, being *l* the sequence length.
4. Select the word *w* starting in the position *p* with length *k*.
5. Count the number of occurrences *c* of the word *w* in the given sequence, allowing for 25% of mismatches.
6. Shuffle *w* into *w*_s_ and count the number of occurrences *c*_*s*_ of the word *w*_*s*_ in the given sequence, allowing for 25% of mismatches.
7. If *c - c*_*s*_ is greater or equal than *c*_*min*_, add the k-mer to the set for the given individual.

#### Evaluation

The fitness calculation is the more crucial step in a GA. It is at this moment when the topic models must be applied and provide a way to obtain solutions for the motif finding problem.

The type of topic model chosen was a correlated topic model (CTM) [7], since it takes into consideration the correlation between topics, and, biologically speaking, motifs also usually show correlation, given that transcription factors which have correlated biological functions bind to them. A CTM makes use of a logistic normal distribution, which, through the transformation of a multivariate normal random variable, allows for a general pattern of variability between the components of the distribution [8]. More specifically, the CTM contained in the R package *topicmodels* [9] was the method used for the construction of the CTM in every iteration.

For each one of the individuals of the population, its set of k-mers, along with the original set of sequences, is fed to a CTM as the vocabulary and the documents respectively. Then the perplexity of the resulting model is measured and returned as the fitness of the given individual.

#### Perplexity

The perplexity of a probabilistic model is a measure of the accuracy with which its distribution predicts a sample. It is the standard used in natural language processing to evaluate the accuracy of the model. The lower the perplexity, the better the model fits the data. The perplexity is calculated by splitting the dataset into two parts: one for training and one for testing, and then measuring the log-likelihood of the unseen documents. As the perplexity is calculated using the corresponding function provided by Hornik and Grün for their CTM implementation, the mathematical formula for perplexity used in this method follows their same definition [9]:

$$ Perp\left(\omega \right)= exp\left\{-\frac{ \log \left(p\left(\omega \right)\right)}{{\displaystyle {\sum}_{d=1}^D}{\displaystyle {\sum}_{j=1}^V}{n}^{(jd)}}\right\} $$

where *n* ^*(jd)*^ refers to the frequency with which the *j*th word appears in the document *d*.

#### Selection

The algorithm tries to give an optimum set of solutions by minimizing the perplexity. Therefore, for the selection of the fittest candidates, an elitist approach is used. In other words, after all of the fitness measurements have been done for a specific generation of individuals, these are selected in random pairs, in which the fittest individual (lower perplexity) survives and the less fit individual is eliminated from the population. After this stage, *N/2* fit individuals remain in the population.

So the next step is generating new individuals by the use of the crossover function to create a new population of *N* individuals.

#### Crossover

The Crossover step is performed after the Evaluation and Selection step to generate new individuals in the population for the next generation.

First, two individuals are randomly selected from the population to act as parents.

The crossover function in this case is a classic one-point crossover in which two children are generated by swapping the data beyond a randomly selected crossover point between both parents. In this case, the crossover point is an index in the array of k-mers of the individuals, which indicates which k-mers to select from each one of the parents (these k-mers are shuffled before this step).

The two newly formed children are added to the population and the process is repeated until the population contains *N* individuals again.

#### Mutation

Mutation happens randomly, according to a parameter that defines the frequency. It is also applied to random individuals. The mutated individual will have a random number of its k-mers slightly shifted from their original position (the position in the sequence randomly increases or decreases by a number no longer than the length of the given k-mer).

#### Post processing

Once the GA is terminated, the fittest individuals are sorted by perplexity (from lower to higher) and selected accordingly as solutions depending on a parameter set by the user that defines how many motifs are expected to be found in the data set. For each one of these solutions, the CTM is generated once again and each one of the resulting topics is returned as a motif of the data set.

### Improving the statistical GA algorithm by the use of the perplexity measurement

The Statistical GA algorithm [5] works as a GA in which the fitness function takes three steps to discard unfit solutions based on three different coefficients [10, 11], in which the main method to select the final candidates is the Mann-Whitney U-Test [12]. Each candidate is a k-mer of a fixed length defined as a parameter represented as a position in a supersequence, which is a concatenation in a random order of all the genetic sequences received as an input. To simplify the calculations, this supersequence is divided in a set of subsequences so that in each iteration the fitness is calculated for a given subsequence and the candidates which show no overrepresentation in the given segment are swiftly discarded without further computations.

#### Adding the use of topic models

**Fig. 2:**
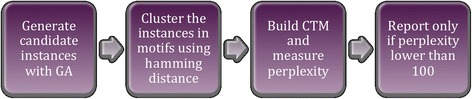
Statistical GA algorithm workflow after including the use of topic models. This figure describes the updated flow of the Statistical GA algorithm after adding the perplexity measurement for the selection of solutions. There are four steps in this flow: the first one, in which the candidate instances are selected by the original Statistical GA; the second one, in which these instances are clustered attending to their similarity calculated by their hamming distance; the third one, which consists of building the CTM and measuring its perplexity, and the last step, which consists of reporting the motif if the perplexity calculated in the previous step is lower than 100

The main drawback of the Statistical GA algorithm is that it reported a big amount of false positives, and one of the main reasons for this was that it had no way to measure the confidence of the results reported. Thus, it reported at least one motif for every data set, ballasting that way the overall performance of the algorithm. The solution here proposed for that problem consists of taking the final set of instances provided by the algorithm, creating a CTM with them, and measuring the perplexity. Then, only in the cases in which the perplexity is lower than certain threshold the motifs returned by the algorithm are reported (Fig. 2). The impact of this was that now the Statistical GA algorithm only reports motifs for those data sets in which there is a CTM that fits well the solutions found. That way, the algorithm is able to measure the confidence of the solutions obtained by the main GA. The threshold was set at 100 for experimental reasons, given that in the tests performed the motifs reported with a perplexity higher than 100 tended to be false positives.

### Assessment

Several studies [1, 2] concluded that evaluating the performance of a motif finding tool has been proven to be a difficult task, and there is no method to compare tools that can give a definitive conclusion about which one is the best and which one is the worst. Keeping this idea in mind, both of the two methods here presented were tested making use of the assessment proposed by Tompa et al. [1] in their study to evaluate the performance of several motif finding tools by the scores obtained in eight different statistical coefficients. It is worth mentioning that only the accuracy of the tools predicting binding sites is evaluated, and other aspects such as the running time of each method, are not measured. The benchmark provided by the assessment, which is the same one used in this study, is formed by 52 data sets, which belong to four different species (fly, human, mouse and yeast) and also 4 negative controls to sum a total of 56 data sets. These 56 data sets are also divided into three different categories: data sets of Type Real, which correspond to the real promoter sequences that contain the original sites that the different tools will try to locate; data sets of Type Generic, which correspond to promoter sequences generated randomly from the same genome, and data sets of Type Markov, which correspond to synthetic sequences generated by a Markov chain. The original assessment compared the efficiency of 14 different tools (Additional file 1: Table S1) [13–26]. Each one of those tools was allowed to report only one (or none) motif per data set. The format in which this motif was reported was as a list of instances and their corresponding positions in the sequences of the data set. Then, the accuracy of how well these instances match the real instances of the motif is studied both at nucleotide and site level. At site level, a predicted site is considered to match the known site if it overlaps at least one quarter of it. With this information, the following eight statistics are used to measure the accuracy of each one of the methods:

- *nSn* (Sensitivity, nucleotide level):
  $$ nSn = \frac{nTP}{nTP+nFN} $$
- *nPPV* (Positive Predicted Value, nucleotide level):
  $$ nPPV = \frac{nTP}{nTP+nFP} $$
- *nSp* (Specificity):
  $$ nSp = \frac{nTN}{nTN+nFP} $$
- *nPC* (Performance Coefficient, nucleotide level) [27]:
  $$ nPC = \frac{nTP}{nTP+nFN+nFP} $$
- *nCC* (Correlation Coefficient) [28]:
  $$ nCC = \frac{nTP \times nTN+nFN \times nFP}{\sqrt{\left(nTP+nFN\right)\left(nTN+nFP\right)\left(nTP+nFP\right)\left(nTN+nFN\right)}} $$
- *sSn* (Sensitivity, site level):
  $$ sSn = \frac{sTP}{sTP+sFN} $$
- *sPPV* (Positive Predicted Value, site level):
  $$ sPPV = \frac{sTP}{sTP+sFP} $$
- sASP (Average Site Performance) [28]:
  $$ sASP = \frac{sSn+ sPPV}{2} $$

The coefficients starting by *n* are statistics at nucleotide level, and the coefficients starting by *s* are statistics at site level. *TP*, *FP*, *TN* and *FN* refer to the number of true positives, false positives, true negatives and false negatives respectively.

Both of the two methods here described were tested using the methodology presented in this assessment and compared to the 14 methods with which it was originally carried out.

## Results

The CTM algorithm was run with the following parameters:

- Motif width between 6 and 30
- Population size: 50
- Number of generations: 90
- Number of instances per individual: 1000
- Maximum number of solutions: 10
- Mutation rate: 0.1

As for the statistical GA algorithm, it was run with the same parameters as in the original study [5]. After adding the perplexity measurement in the post processing stage, a new restriction was included: Only the motifs reported with a perplexity lower than 100 were considered as solutions.

All of the tests were run in a laptop computer with a 2.6 GHz Intel Core i5 processor and an 8 GB 1600 MHz DDR3 memory.

**Fig. 3:**
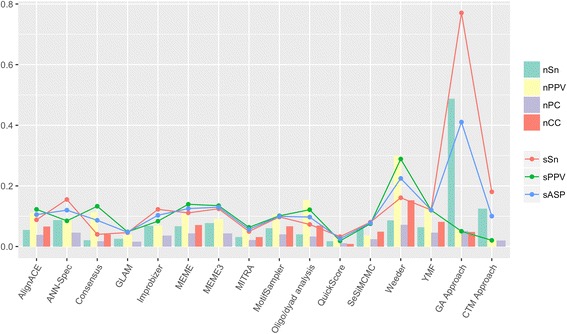
Average statistical values for all 56 data sets. This figure shows the average scores obtained by each one of the tools studied for each one of seven different statistics for all the 56 data sets of the benchmark. The Statistical GA method is shown as GA approach, and the CTM method as CTM approach

**Fig. 4:**
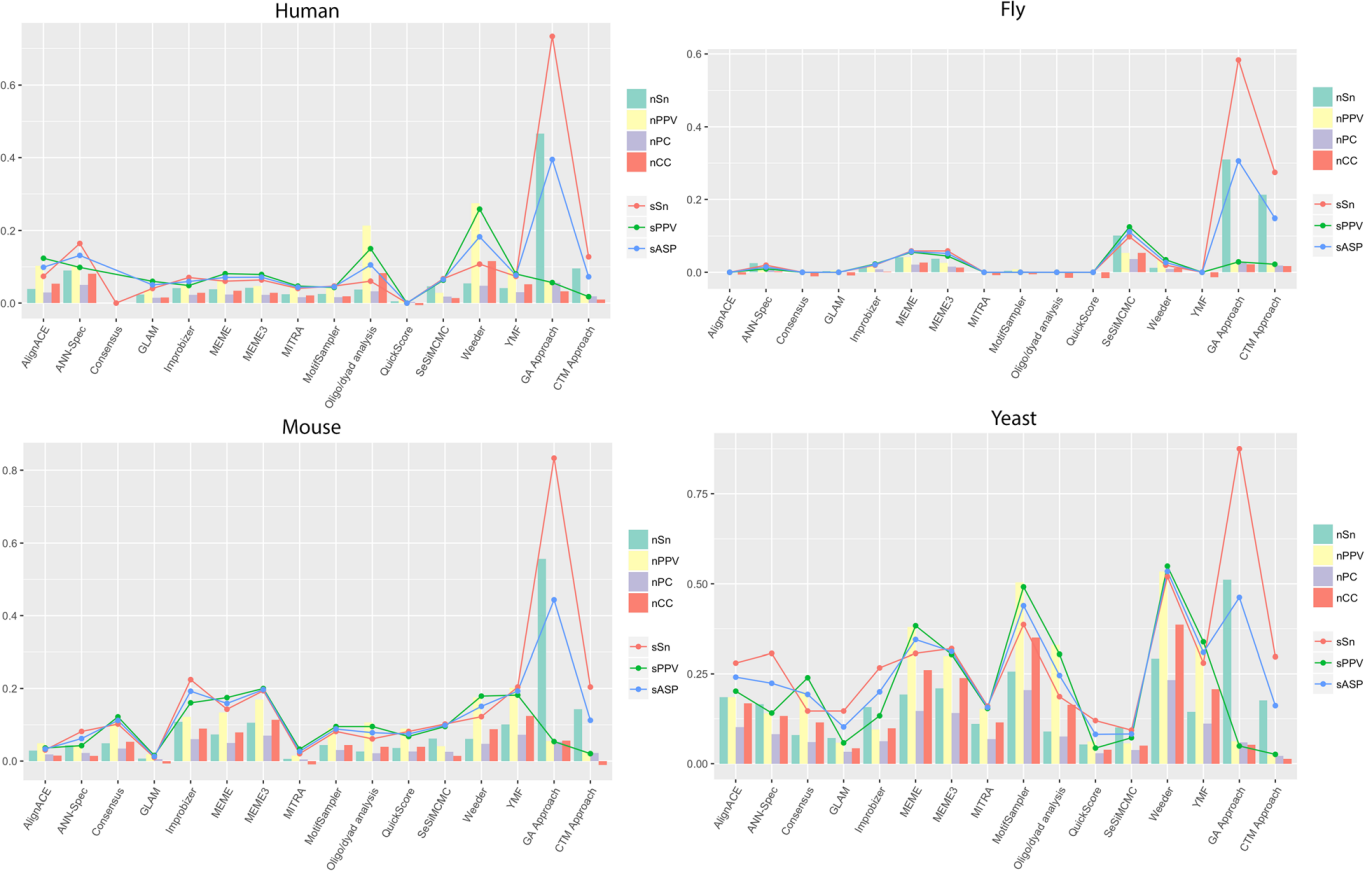
Average statistical values for each organism. This figure shows the average scores obtained by each one of the tools studied for each one of seven different statistics grouped by the four different species contained in the data sets. The Statistical GA method is shown as GA approach, and the CTM method as CTM approach

Figure 3 summarizes the average values of the statistics previously defined for each one of the 14 tools originally analyzed in the assessment and for both of our proposed tools. Figure 4 shows the average values grouped by organisms.

To calculate the average values, we followed the same process as in the original assessment. In a first step, the values of *nTP*, *nFP*, *nFN*, *nTN*, *sTP*, *sFP* and *sFN* obtained for each one of the data sets are summed. Then, each one of these summed values is considered as the given score of a large data set, and the eight statistics are calculated for that large data set , obtaining that way the average scores.

## Discussion

As previously stated, none of the statistics analyzed should ever be taken as an absolute measurement of the quality of the methods. The authors of the assessment [1] themselves indicate several factors that affect the results and might give a wrong impression about the performance of the different algorithms:

- This assessment, as any other possible method, can never be considered a standard method to measure the biological significance of the studied tools, since it is still unknown how the subjacent biology works.
- As each one of the algorithms was required to predict only one (or none) motif for each data set, there might be an arbitrary component in the candidate selected by each tool.
- The assessment requires each tool to report only one (or none) motif for each data set. However, it is known that, especially in the case of the data sets of Type Real, they are likely to contain more than one motif.
- Many of the known binding sites are longer than 30 bp. Our tools, as well as most of the others, were run for motifs no longer than 30 bp in the case of the CTM algorithm and 12 bp in the case of the statistical GA algorithm. This affects the performance at nucleotide level even if the performance at site level is high.
- The assessment relies on TRANSFAC [29] as its only source of known binding sites. As the information obtained from TRANSFAC is not contrasted with other sources, it might as well contain errors.
- The above explained method used to compute the average scores of every tool tends to penalize those tools that make wrong predictions more than those that make no predictions at all, as 0 is the default value for the cases in which no motifs are reported.

As long as all these factors are not forgotten, some important conclusions regarding the performance of the different methods can still be inferred from the use of the benchmark proposed in the assessment.

First of all, the CTM method shows levels in Sensitivity (both at nucleotide level, *nSn*, and at site level, *sSn*) only outperformed by our other method, the Statistical GA (Figs. 3 and 4). It also shows a remarkable Average Site Performance (*sASP*) and, regarding the rest of statistics, even though the numbers obtained are not especially satisfying, they are comparable to most of the other methods.

Thus, we can already reach the conclusion that topic models are a perfectly valid method to design motif finding algorithms.

**Fig. 5:**
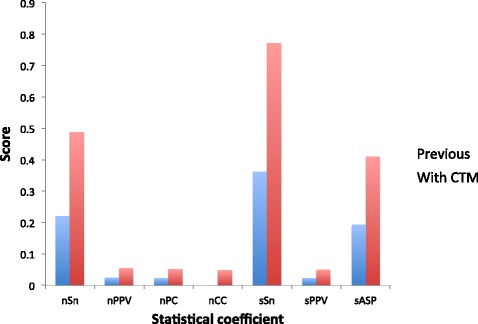
Comparison of statistics for the Statistical GA method before and after filtering by perplexity. Here it is shown the improvement in the average scores of the Statistical GA method for seven different statistics obtained by the addition of the filtering of the results by their perplexity in the corresponding CTM

As for the Statistical GA method, Fig. 5 shows the improvement in all of the average statistics after narrowing down the results reported according to the perplexity shown in the CTM. All of the scores for the different statistics are practically doubled after filtering out the motifs for which the perplexity of the corresponding CTM is higher than 100.

This tool now clearly outperforms most of the other methods, showing levels of *nSn*, *sSn*, and *sASP* to which any of the other tools can hardly be compared (Figs. 3 and 4). This further proves the usefulness of topic models for motif discovery tools.

Given the nature of both methods, and the high number of true positives shown (especially at site level), it seems clear that both succeed in predicting many of the sites but lack of a mechanism to detect false positives. In other words, as the high scores in Sensitivity and Average Site Performance show, both methods can correctly report most of the known motifs, but they locate too many instances of them in the input sequences, so that the number of false positives reported in the assessment, especially at nucleotide level, appears too large, in spite of the correctness of the consensus or the score matrix given by the algorithms as a result. We therefore believe that the high number of false positives is due, to a large extent, to the nature of the assessment. This drawback was considerably reduced in the new version of the statistical GA algorithm, thanks to the use of the perplexity measurement to avoid predicting wrong motifs, but we believe the number of false positives in the assessment could still be shortened if some sort of method was used in the post processing step to obtain only the correct instances of the known site by the use of a weight matrix based on the consensus sequence, instead of simply reporting all of the candidate instances found as both tools currently do. For the CTM method, a way to filter out the results which are not reported with high confidence is required as well.

The method that gives the best overall statistics after the Statistical GA method is Weeder. As the authors of the assessment clarify [1], one of the main reasons for that is the way in which it was run. The author of the tests decided to pick a cautious mode, that is, to predict a motif only if there is a high confidence of its existence. That explains, therefore, the great improvement in the statistics for our Statistical GA tool, and that it is mostly due to the way to calculate the average statistics proposed in the assessment.

As for the running time, as stated before, it is not an object of the assessment, which is focused on the accuracy of the sites predicted. However, it is worth mentioning that the CTM method slows down considerably when the number of input sequences is bigger than three. Therefore, some solution for this problem, such as dividing the data sets into subgroups of three or fewer sequences, will be required. The Statistical GA method, on the other hand, is able to report the results of data sets of any size in a matter of minutes.

## Conclusions

DNA motif finding still remains as one of the most challenging tasks for researchers, and so it is the task of comparing the performance of the different existing tools, given that each one of them has been designed using very heterogeneous algorithms and models, and that there is still little known about the subjacent biology. Therefore, we must insist on the fact that nowadays it is impossible to define a standard quality measurement to evaluate the performance of the different tools.

Most of the studies on the performance of motif finding algorithms [2] conclude

that the best option for biologists to try to predict sites in a set of sequences is never to rely on a single tool, but better to use a few complementary tools and combine the top predicted results of each one of them.

In line with this, we believe that the methods here described, despite their drawbacks, can perfectly be part of those tools that biologists use in combination with others to predict *de novo* binding sites in sets of biological sequences. Especially in the case of the CTM method, there are still many improvements to be done. However, given the results, it can already be used as a ground for future tools based on the use of topic models as a reliable method for motif finding.

## Additional file

Additional file 1: This table shows the tools that were studied in the original assessment by Tompa et al. [1] and the two methods presented in this study, each one with a short description of their underlying methodologies. (DOCX 15 kb)

## Abbreviations

ASP: Average site performance
CTM: Correlated topic model
FN: False negative
FP: False positive
GA: Genetic algorithm
TFBS: Transcription factor binding site
TN: True negative
TP: True positive
