# Supplementary material for: A study on the application of topic models to motif finding algorithms
Source: BMC Bioinformatics. 2016 Dec 22;17(Suppl 19):502. doi: 10.1186/s12859-016-1364-3 (PMC5259985; doi:10.1186/s12859-016-1364-3)
Supplement: Additional file 1: — This table shows the tools that were studied in the original assessment by Tompa et al. [1] and the two methods presented in this study, each one with a short description of their underlying methodologies. (DOCX 15 kb) [file 12859_2016_1364_MOESM1_ESM.docx]

## Additional file 1: Table S1 - Motif finding tools of the assessment

This table shows the tools that were studied in the original assessment by Tompa et al. [1] and the two methods presented in this study, each one with a short description of their underlying methodologies.

| Tool | Methodology | Reference |
| --- | --- | --- |
| AlignACE | It is based on Gibbs Sampling, and uses a log likelihood score to measure the level of overrepresentation. | 16 |
| ANN-Spec | It uses a weight matrix to model the DNA binding specificity of each TFBS. | 17 |
| Consensus | It uses weight matrices to model the motifs and then tries to find which matrix has more information content. | 18 |
| GLAM | It is based on Gibbs Sampling, and it optimizes the motif width of each alignment automatically. | 19 |
| The Improbizer | It makes use of expectation maximization to try to find the weight matrices whose presence is statistically improbable. | 20 |
| MEME | It uses expectation maximization to optimize the E-value of the information content of each TFBS. | 21 |
| MEME3 | MEME3 tries to improve MEME by using a correction factor in the objective function that boosts its accuracy. | 21 |
| MITRA | It makes use of the hypergeometric score of the occurrences of each candidate motif in the input sequences in contrast to a set of background sequences. | 22 |

| Tool | Methodology | Reference |
| --- | --- | --- |
| MotifSampler | It is based on a combination of Gibbs Sampling and a Markov model. | 23 |
| Oligo/dyad-analysis | It counts how many occurrences of each k-mer appear in the sequences in contrast with the expectation of a negative binomial distribution. | 24, 25 |
| QuickScore | It is based on exhaustive searching and a background Markov model to detect if k-mers are rare or frequent. | 26 |
| SeSiMCMC | It uses a modification of Gibbs sampling, combined with a Markov model. | 27 |
| Weeder | It performs an exhaustive oligo frequency analysis to then select instances by a consensus-based algorithm. | 28 |
| YMF | It is based on an exhaustive search in which the motifs with the highest z-score are selected. | 29 |
| GA Approach | It is based on a genetic algorithm and three different statistical coefficients to measure which k-mers occur unusually frequently. | 5 |
| CTM Approach | It is based on the use of a CTM and the structure of a genetic algorithm to select clusters of k-mers that have a low perplexity. |  |
